# Spatial and Temporal Variation of Urban Heat Islands in French Guiana

**DOI:** 10.3390/s24061931

**Published:** 2024-03-18

**Authors:** Gustave Ilunga, Jessica Bechet, Laurent Linguet, Sara Zermani, Chabakata Mahamat

**Affiliations:** ESPACE-DEV (Espace pour le Développement), University of La Réunion, University of Montpellier, Institut de Recherche pour le Développement (IRD), University of Antilles, University of French Guiana, 97300 Cayenne, France; ilunga.gus@gmail.com (G.I.); laurent.linguet@univ-guyane.fr (L.L.); sara.zermani@univ-guyane.fr (S.Z.); chabakata.mahamat@univ-guyane.fr (C.M.)

**Keywords:** surface urban heat island, land surface temperature, remote sensing, French Guiana

## Abstract

A surface urban heat island (SUHI) is a phenomenon whereby temperatures in urban areas are significantly higher than that of surrounding rural and natural areas due to replacing natural and semi-natural areas with impervious surfaces. The phenomenon is evaluated through the SUHI intensity, which is the difference in temperatures between urban and non-urban areas. In this study, we assessed the spatial and temporal dynamics of SUHI in two urban areas of the French Guiana, namely Ile de Cayenne and Saint-Laurent du Maroni, for the year 2020 using MODIS-based gap-filled LST data. Our results show that the north and southwest of Ile de Cayenne, where there is a high concentration of build-up areas, were experiencing SUHI compared to the rest of the region. Furthermore, the northeast and west of Saint-Laurent du Maroni were also hotspots of the SUHI phenomenon. We further observed that the peak of high SUHI intensity could reach 5 °C for both Ile de Cayenne and Saint-Laurent du Maroni during the dry season when the temperature is high with limited rainfall. This study sets the stage for future SUHI studies in French Guiana and aims to contribute to the knowledge needed by decision-makers to achieve sustainable urbanization.

## 1. Introduction

Urbanization is rapidly occurring due to increased global population and rural exodus [1,2]. In 2018, it was estimated that 55% of the global population lived in urban areas, and this number is expected to rise to 60% by 2030 [3]. In the process of urbanization, natural and semi-natural surfaces, such as vegetation cover, natural ponds, and raw lands, are replaced by impervious surfaces like buildings, pavements, and roads [4,5]. These changes disturb the albedo and the energy balance and alter the thermal properties of urban areas [6], leading to the phenomenon referred to as a surface urban heat island (SUHI).

The SUHI is a phenomenon whereby temperatures in urban areas are significantly higher than those of surrounding rural and natural areas [7,8]. It is, for example, estimated that between 2003 and 2005, the average daily temperature in summer in urban areas of more than 500 km^2^ rose by 4.7 °C [6]. Another study conducted in the South of Brazil found the difference in surface temperature between the urbanized core and the urban edge in the summer of the city of Rio de Janeiro to be up to 10 °C [9]. Nevertheless, the intensity of the SUHI depends on several factors, such as regional climate and weather, the type of urbanization, the transportation system, the type of materials used in construction, and the presence of vegetation in urban areas [7].

Due to its potential health and environmental risks, the SUHI is becoming one of the most pressing urban-related environmental issues [10] and a major concern for urban planners [11]. Increasing urban temperature can result in an increased frequency of heat waves, atmospheric pollution, and health problems [12,13,14]. Furthermore, the SUHI can increase mortality rates due to urban heat stress [10]. The SUHI can also increase energy and water consumption [14,15]. As a result, the SUHI phenomenon has gained the attention of researchers, and an increasing number of related studies have been conducted in various cities across the world [6].

The SUHI is identified by assessing temperature data. The two sources of temperature data that are generally used to study the SUHI are air temperature measurements and remote sensing land surface temperature (LST) [16]. Air temperatures are direct SUHI measures but are usually only available for single measurement stations or traverses through a city [16]. Moreover, this approach can be time-consuming when covering large areas, and few collection points may not reflect the great heterogeneity of urban areas. On the other hand, remote sensing technologies such as satellites offer the opportunity to collect LST data over large areas at various spatiotemporal resolutions [17]. Many studies on SUHI have demonstrated the added value of remote sensing in studying the SUHI phenomenon [18,19,20]. Examples of satellites (with thermal imagery capability) used in SUHI studies include a Landsat [10], Moderate-Resolution Imaging Spectroradiometer (MODIS) [9], Advanced Spaceborne Thermal Emission and Reflection Radiometer (ASTER), and Sentinel-3.

Several studies have been conducted in various South American countries using LST data and air temperature measurements [21,22,23,24,25,26]. All studies show that the SUHI phenomenon is higher during hotter seasons. In [22], the authors make use of 16 Landsat images from the period of 2008–2011 to detect the surface urban heat islands on a Brazilian coastal region; this allowed them to make coarser identification of SUHI-dependent factors, hence a negative correlation between LST and the normalized vegetation index and positive correlation between LST and the normalized built-up index. The authors also highlighted that the urbanization was performed inadequately and brings environmental and societal questions. However, their study does not allow to understand the spatiotemporal evolution of the SUHI. The authors in [23] make use of 16 years of MODIS images to evaluate spatially and temporally the SUHI in 21 coastal and inland cities of Brazil. The regions of interest have high population density. This study brings new insights for the SUHI study in South America and shows a relation between built density and a high SUHI at nighttime. 

French Guiana is a French outermost region located in South America with neighboring countries Brazil and Suriname. The territory has an equatorial climate, with average annual temperatures of 26 °C and abundant rainfall. Its particularity is that it is a sparsely inhabited territory, of which the Amazonian forest covers 96% of its surface area. Hence, French Guiana has a smaller population density than its neighbor, Brazil. Even though there are temperature differences between urban and non-urban areas, that can be high, and the region already has a significant urbanization phenomenon. 

In this study, which is the first one to be conducted in French Guiana, we are assessing the spatial and temporal variation of the SUHI in two of the largest urban areas of French Guiana for the year 2020. Here, we propose using MODIS-based gap-filled LST data to conduct our assessment. As the MODIS satellite provides two images per day (one at daytime and one at nighttime), it is a suitable choice to conduct this study. Indeed, even if Landsat satellites provide images with a higher resolution, images are provided only once every 16 days. Moreover, the cloud cover issue is crucial for the SUHI study; hence, we have chosen a gap-filled dataset that allows us to counter this issue.

The two urban areas under study are Ile de Cayenne and Saint-Laurent du Maroni (hereafter Saint-Laurent), two of the largest urban areas in French Guiana, which houses more than 50% of the total French Guiana population. Due to their high rate of urbanization, these areas are prone to the SUHI and its effects. Therefore, it is critical to assess the dynamics of the SUHI in these urban areas and provide decision-makers with much-needed information to achieve sustainable urbanization. Moreover, Ile de Cayenne is a coastal area, and Saint-Laurent is an inland one. 

This study aims to evaluate the spatiotemporal evolution of the SUHI in the two more urbanized areas of French Guiana. The influence of seasons and time (daytime and nighttime) will be assessed using a gap-filled dataset. The SUHI intensity and the urban thermal filed variance index (UTFVI) will be used to detect the presence of SUHIs and evaluate their distribution. The UTFVI is a common score used to detect local differences in temperature. Moreover, the relations between the SUHI and land cover, elevation, and distance to the closest water point are assessed, allowing for an enhanced understanding of the SUHI repartition.

## 2. Materials and Methods

### 2.1. Study Area

**Figure 1:**
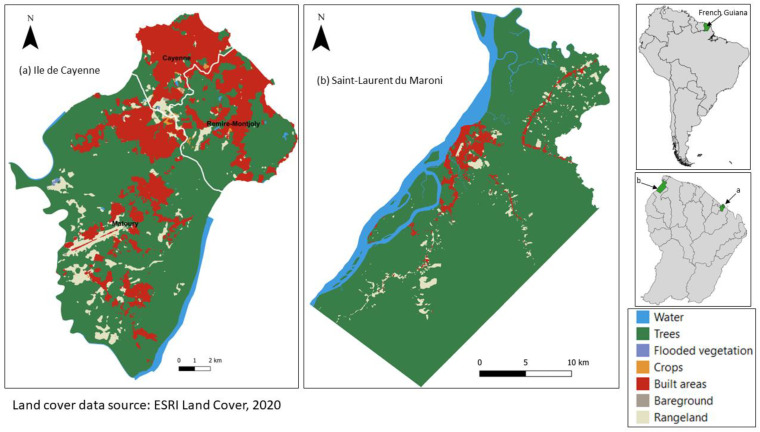
Landcover of the study area. Source: ESRI, 2020 [31].

French Guiana is an overseas department of France located on the northeast coast of South America (Figure 1). French Guiana has a humid equatorial climate with an average annual temperature of 26 °C; precipitation ranges between 2000 mm and 4000 mm per annum [27]. French Guiana is characterized by four seasons of uneven durations: a short rainy season from mid-November to late January; a short dry season—also called the”little summer of March”—between early February and mid-March; a long rainy season from late March to late July; and a long dry season from late July to mid-November [28]. Hence, the seasons last, respectively, around 77, 43, 138, and 107 days for a standard year. Wind direction in French Guiana is predominantly eastward, more precisely northeast (during the rainy season) and southeast (during the dry season) [29].

Ile de Cayenne and Saint-Laurent are two of the largest urban areas of the French Guiana (Figure 1). Ile de Cayenne is the name given to the area occupied by the municipalities of Cayenne, Matoury, and Remire-Montjoly. Ile de Cayenne has a surface area of 206.91 km^2^ with a population of 122,737 [30]. Saint-Laurent, on the other hand, is a municipality with a surface area of 4830 km^2^ and a population of 45,576 [30]. The city of Saint-Laurent is built around the Maroni River. Combined, the population of these two urban areas makes up more than 50% of the total population of French Guiana. Saint-Laurent has a large surface predominantly covered by forest. To better study the SUHI phenomenon, we only selected a subset of the municipality with a high concentration of built-up areas.

### 2.2. Land Surface Temperature (LST) Data

LST data are used to assess the SUHI phenomenon. LST data are acquired through optical satellites at different spatial and temporal resolutions. However, the presence of clouds poses a significant challenge when working with images acquired from those satellites. Due to its geographical location, French Guiana is frequently covered by clouds. As a result, producing a comprehensive LST time series remains challenging. Nevertheless, recent advances in cloud removal and gap-filling techniques offer the opportunity to reconstruct satellite images with various levels of precision. 

In this study, we are using a MODIS-based gap-filled LST dataset produced by [32]. The dataset offers daily daytime (01 h 30 pm UTC ) and nighttime (01 h 30 am UTC) LST data with a spatial resolution of 1 km covering the years 2003 to 2020. However, in this study, we worked with the 2020 time series with the objective of understanding SUHI dynamics in our study area during this year. This dataset is freely accessible on the Iowa State University’s DataShare (Please follow the link hereafter: https://doi.org/10.25380/iastate.c.5078492 [33]). Working with this dataset is worthwhile because of its high accuracy and the removal of the cloud cover issue. Indeed, as reported in [32], these data have an average root-mean-squared error of less than 2° for daytime and less than 1.5° for nighttime. Moreover, this gap-filled dataset allows us to overcome the cloud cover issue, a significant problem in studies concerning satellite images, especially in French Guiana, located in the intertropical zone with a high cloud presence.

In this study, we are using a MODIS-based gap-filled LST dataset produced by [32]. The dataset offers daily daytime (01 h 30 pm UTC ) and nighttime (01 h 30 am UTC) LST data with a spatial resolution of 1 km covering the years 2003 to 2020. However, in this study, we worked with the 2020 time series with the objective of understanding SUHI dynamics in our study area during this year. This dataset is freely accessible on the Iowa State University’s DataShare (Please follow the link hereafter: https://doi.org/10.25380/iastate.c.5078492 [33]). Working with this dataset is worthwhile because of its high accuracy and the removal of the cloud cover issue. Indeed, as reported in [32], these data have an average root-mean-squared error of less than 2° for daytime and less than 1.5° for nighttime. Moreover, this gap-filled dataset allows us to overcome the cloud cover issue, a significant problem in studies concerning satellite images, especially in French Guiana, located in the intertropical zone with a high cloud presence.

### 2.3. LST Spatial Distribution

To assess the LST spatial distribution, we first normalized the LST data in order to facilitate LST comparison across different days. We normalized the data using the following formula:

(1) $$\mathrm{LST}\;\mathrm{normalisation}=\frac{\mathrm{LST}-T_{\min}}{{\mathrm{LST}}_{\max}-{\mathrm{LST}}_{\min}}$$

where LST_min_ is the minimum LST, and LST_max_ is the maximum LST.

### 2.4. Maximum Daytime and Nighttime LST Temporal Trend

According to the literature, high LST values are usually observed in urban areas. Therefore, identifying daily maximum daytime LST and maximum nighttime LST can provide insight into the cooling rate of urban areas. The difference between maximum daytime LST and maximum nighttime LST, which we will call here delta LST, was calculated as follows:

(2) ΔLST=maxdaytime LST−max nighttime LST

### 2.5. Assessment of the SUHI Intensity

The SUHI intensity is defined here as the difference between urban LST and non-urban LST. This assessment allows us to quantify how much hotter the urban area is compared to non-urban areas. To determine the SUHI intensity, we first reclassified land cover into two broad categories: urban and non-urban. According to the land cover given in Figure 1, built areas are classified as urban, and vegetation and bare ground are classified as non-urban. Hence, the hereafter denominated urban LST is computed as the average value of all LST values of pixels classified as urban (i.e., corresponding to the land cover “built area”); likewise, the hereafter denominated non-urban LST is computed as the average of all LST values of pixels classified as non-urban (i.e., corresponding to all other land cover categories except “water”). Using all the LST values of a category allows us to reduce the uncertainties introduced by the choice of a sample point in each category. We then proceeded to calculate the daily daytime and nighttime average LST for each category. Lastly, we applied the following formula to determine the daily SUHI intensity:

(3) SUHI intensity=urban LST−nonurban LST

with urban LST as the average LST of the urban fabric and non-urban LST as the average LST of the non-urban fabric.

Let us also introduce the SUHI_p_ intensity, which is the evaluation of SUHI at each pixel; that is, for each pixel p in the image,

(4) $${\mathrm{SUHI}}_{p}\;\mathrm{intensity}={\mathrm{LST}}_{p}-\mathrm{non}-\mathrm{urban}\;\mathrm{LST}$$

where LST_p_ is the LST value at pixel p and non-urban LST is the mean value of all non-urban pixels (as defined previously).

To summarize, in this document, when mentioning SUHI intensity, the authors refer to Equation (3), and SUHI_p_ refers to Equation (4).

### 2.6. Assessment of the Urban Thermal Filed Variance Index (UTFVI)

The UTFVI is an index commonly used to identify areas experiencing the SUHI phenomenon. The UTFVI is calculated from LST using the following equation [16]:

(5) $$\mathrm{UTFVI}=\frac{\mathrm{LST}-{\mathrm{LST}}_{\mathrm{mean}}}{\mathrm{LST}}$$

The UTFVI score is higher for pixels that are warmer than their surrounding areas. It is, therefore, a good tool to identify hotter areas, i.e., those subject to the SUHI. The UTFVI score is further classified based on a reference table (Table 1) to determine the SUHI phenomenon of the area.

### 2.7. Linear Correlation between the LST and Influencing Factors

The proximity to water, elevation, and land cover are cited factors in the literature that influence the land surface temperature [34,35] and the SUHI intensity.

It is important to point out that the MODIS LST product is provided for land regions: there is no estimation for large surfaces of water (big lakes, oceans, etc.). This ensures that the influence of water bodies on the provided temperature is avoided. Moreover, the reader may keep in mind that when talking of water in this study, only inland waterways are considered, i.e., the Cayenne River at the west and the Mahury estuary at the south of Ile de Cayenne and the Maroni River for Saint-Laurent. 

This study explores which factors significantly influence the SUHI_p_ according to a linear dependence. Moreover, a linear relation is formulated as:

$${\mathrm{SUHI}}_{p}=a+b_{w}\times {\mathrm{dist}}_{w}+b_{e}\times \mathrm{dem}+b_{l}\times \mathrm{land}\;\mathrm{cover}$$

where a is the intercept, $b_{w}$, $b_{e}$, and $b_{l}$ are, respectively, the slope of the normalized distance of the pixel to the closest water point, the slope of the normalized digital elevation model, and the slope of the normalized land cover type; ${\mathrm{dist}}_{w}$ is the normalized distance to the closest water point, dem the normalized digital elevation model, and land cover is either water, urban or non-urban. The original digital elevation model map has been provided by [36]. The normalization $\bar{F}$ of each factor F, has been proceeded following:

(6) $$\bar{F}=\frac{F-F_{\min}}{F_{\max}-F_{\min}}$$

With $F_{\min}$ and $F_{\max}$, respectively, the minimal and maximal value of the considered factor. 

## 3. Results

For illustration purposes, in Section 3 and Section 4, we selected four dates representing French Guiana’s four seasons. These dates are 1 February 2020 (the beginning of the short dry season), 1 April 2020 (the beginning of the long rainy season), 1 August 2020 (the beginning of the long dry season), and 1 December 2020 (the beginning of the short rainy season). Moreover, daytime and nighttime data are presented.

It is important to point out that missing pixels in the proposed figures correspond to an absence of information in the original gap-filled dataset. 

### 3.1. LST Spatial Distribution

**Figure 2:**
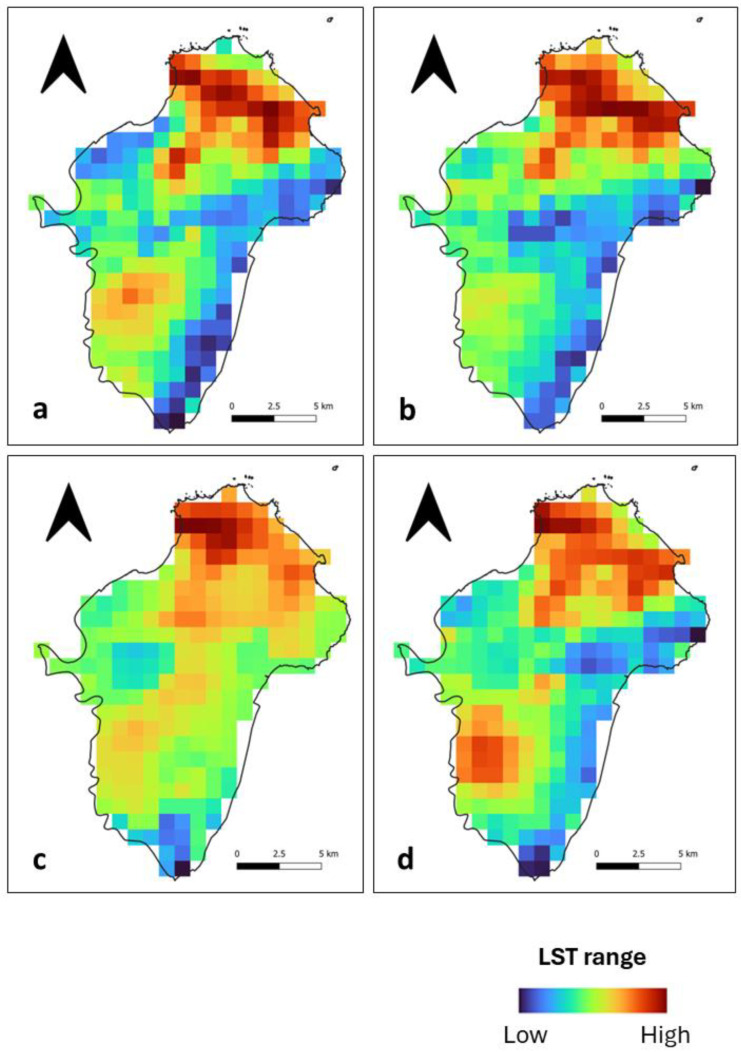
Ile de Cayenne normalized daytime LST for (**a**) 1 February 2020 (**b**) 1 April 2020 (**c**) 1 August 2020 and (**d**) 1 December 2020.

**Figure 3:**
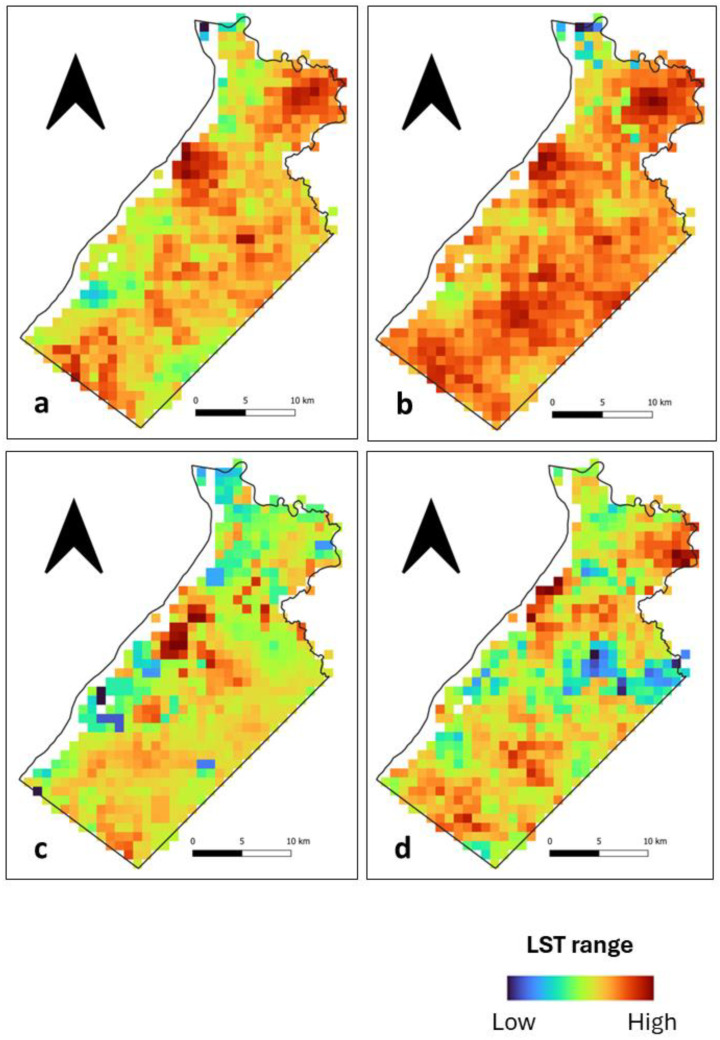
Saint-Laurent normalized daytime LST for (**a**) 1 February 2020 (**b**) 1 April 2020 (**c**) 1 August 2020 and (**d**) 1 December 2020.

During the day, we observed that high LST patches at Ile de Cayenne (Figure 2) are located in the north and, to some extent, in the southwest. Their location corresponds to where there is a concentration of build-up areas (Figure 1). Mild to low LSTs are distributed across the study area. At Saint-Laurent (Figure 3), we have an important concentration of high LST toward the northwest of the municipality, which is the location of the city center. We can also notice high LST in the northeast. The remaining municipality displays mild to low LST patches.

**Figure 4:**
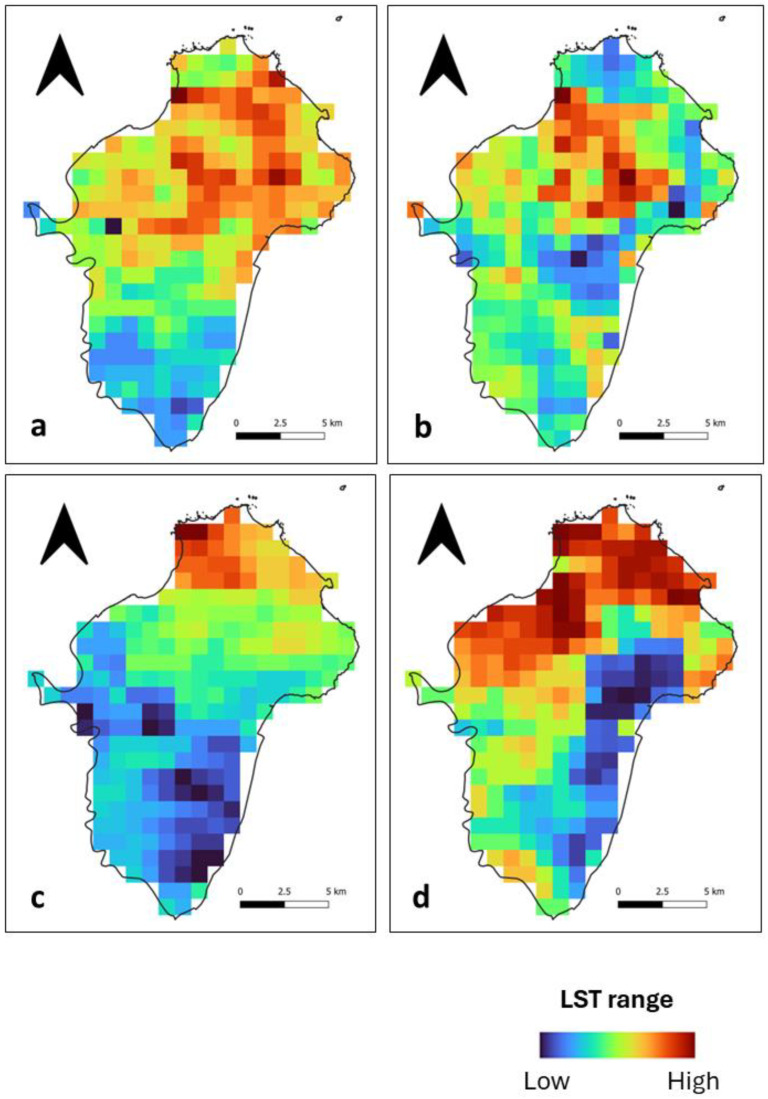
Ile de Cayenne normalized nighttime LST for (**a**) 1 February 2020 (**b**) 1 April 2020 (**c**) 1 August 2020 and (**d**) 1 December 2020.

**Figure 5:**
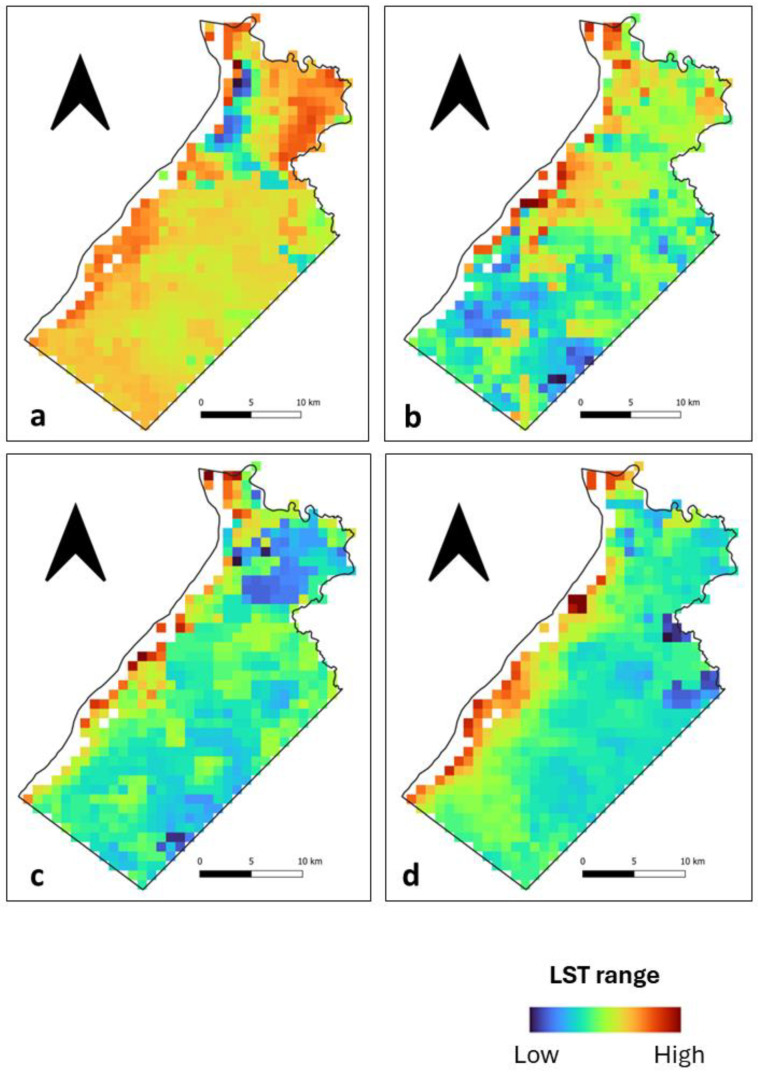
Saint-Laurent normalized nighttime LST for (**a**) 1 February 2020 (**b**) 1 April 2020 (**c**) 1 August 2020 and (**d**) 1 December 2020.

Nighttime spatial LST distribution at both Ile de Cayenne (Figure 4) and Saint-Laurent (Figure 5) shows an apparent shift or expansion of high LST from their daytime location. At Ile de Cayenne, the displacement is generally toward the west of the region, while at Saint-Laurent, high LST is predominantly observed in the west, extending south-north along the Maroni River. At Ile de Cayenne, for images representing long rainy and long dry seasons, there is no shift of the location of high LST, contrary to the case of short seasons, while a shift is present for all seasons for Saint-Laurent.

Furthermore, despite the general trend observed, we can notice some differences in the spatial distribution of LST between days, whether during the daytime or nighttime. This can be attributed to the weather and also to the season.

### 3.2. Daytime and Nighttime Maximum LST Temporal Trend

**Figure 6:**
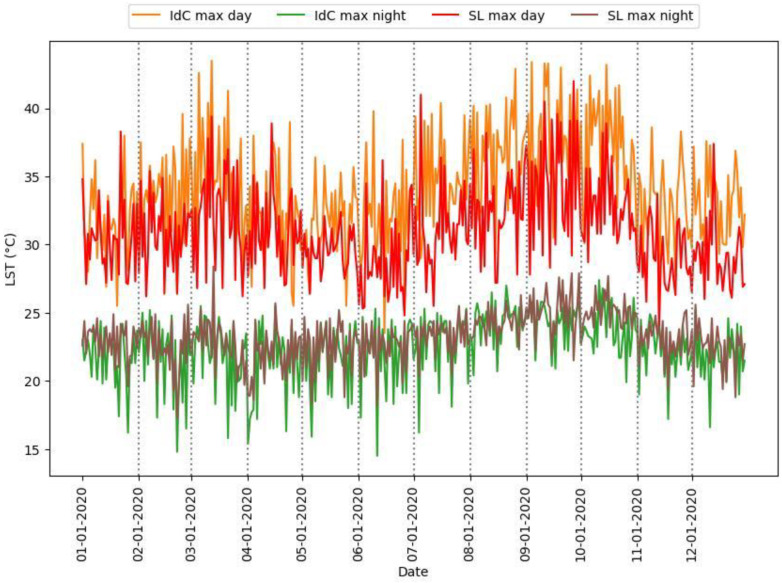
Daytime and nighttime maximum LST.

**Figure 7:**
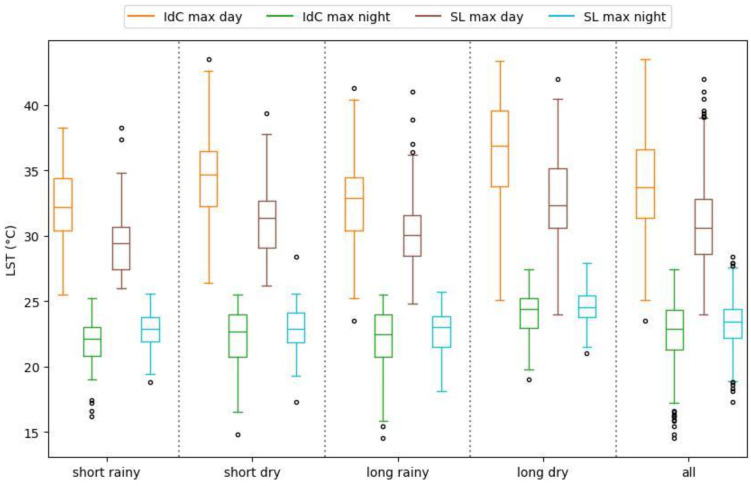
Box plot of daytime and nighttime maximum LST.

**Figure 8:**
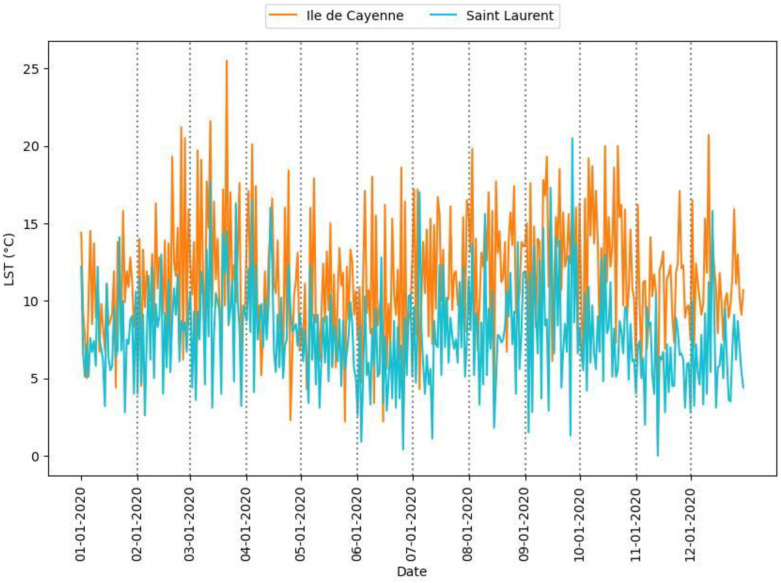
Delta maximum LST.

In this section, we present our findings about the occurrence of daytime and nighttime maximum LST (Figure 6, Figure 7 and Figure 8 and Table 2). Figure 6 presents the maximum LST values for daytime and nighttime throughout the year for Ile de Cayenne and Saint-Laurent. Figure 7 represents the maximum daytime and nighttime LST boxplot for the two regions. Figure 8 represents the ΔLST for Ile de Cayenne and Saint-Laurent. Table 2 presents the average values of maximum LST, ΔLST, mean urban and non-urban LST, and SUHI intensity for Ile de Cayenne and Saint-Laurent year-round and each season for all daytimes and nighttimes.

The average daily maximum LST at Ile de Cayenne is 34 °C at daytime and 22 °C at nighttime. At Saint-Laurent, the average daily maximum LST at daytime is 31 °C and 23.2 °C at nighttime. The average delta maximum LST sits at 11.5 °C for Ile de Cayenne and 6.8 °C at Saint-Laurent. Furthermore, we have observed that maximum daytime LST both at Ile de Cayenne and Saint-Laurent peaks around the month of March, during which French Guiana experiences a “little summer”, and again between August and October, corresponding to the dry season known for being hot with limited rainfall events. Conversely, maximum nighttime LST tends to be constant throughout the year but shows a slight increase between September and October. Nevertheless, as shown in Figure 7, Ile de Cayenne appears to have a higher LST than Saint-Laurent. Moreover, LST values at Saint-Laurent are more constant throughout the seasons than at Ile de Cayenne.

The average ΔLST for Saint-Laurent is smaller than for Ile de Cayenne and tends to be higher in dry seasons than rainy ones for the two regions. 

### 3.3. Assessment of SUHI Intensity

**Figure 9:**
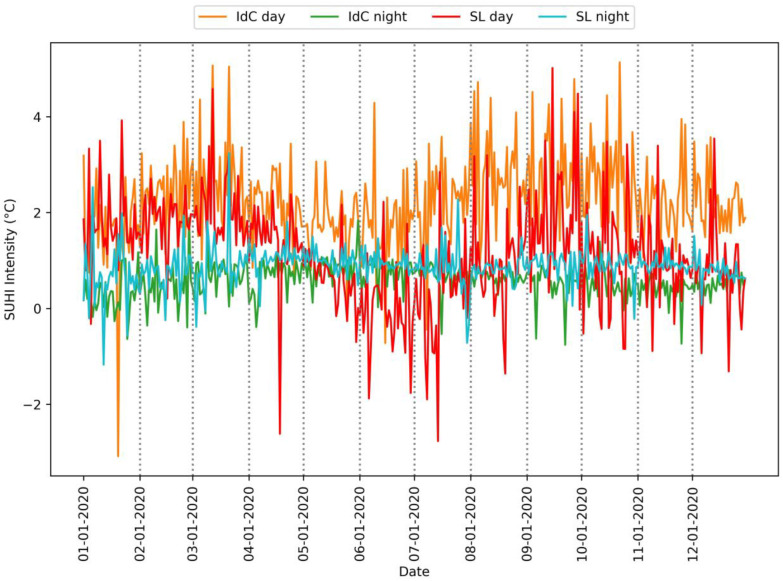
Daytime and nighttime SUHI intensity for Ile de Cayenne and Saint-Laurent.

This section presents the results of the daily SUHI intensity (Figure 9). At Ile de Cayenne, the daytime average SUHI intensity is 2.6 °C while the nighttime average sits at 0.6 °C. We also observed peaks of high SUHI intensity, notably on 22 October 2020, where the daytime SUHI intensity was 5.1 °C, and on 28 February 2020, where the nighttime SUHI intensity was 1.9 °C. At Saint-Laurent, the daytime average SUHI intensity was 1.2 °C, and the nighttime average was 0.8 °C. Regarding the peaks of high SUHI intensity, the highest daytime intensity was 5 °C on 15 September 2020, and the highest nighttime intensity was 3.2 °C on 21 March 2020.

Generally, for both Ile de Cayenne and Saint-Laurent, particularly at nighttime, the SUHI intensity was generally lower than 2 °C, suggesting an important cooling effect on urban areas. However, we have observed negative SUHI intensity, implying that urban areas were cooler than surrounding non-urban areas. Given the context of our study areas, we suspect a potential error in the data, whereby non-urban areas are essentially forest cover. Furthermore, SUHI intensity at Ile de Cayenne appears to be higher than at Saint-Laurent.

**Figure 10:**
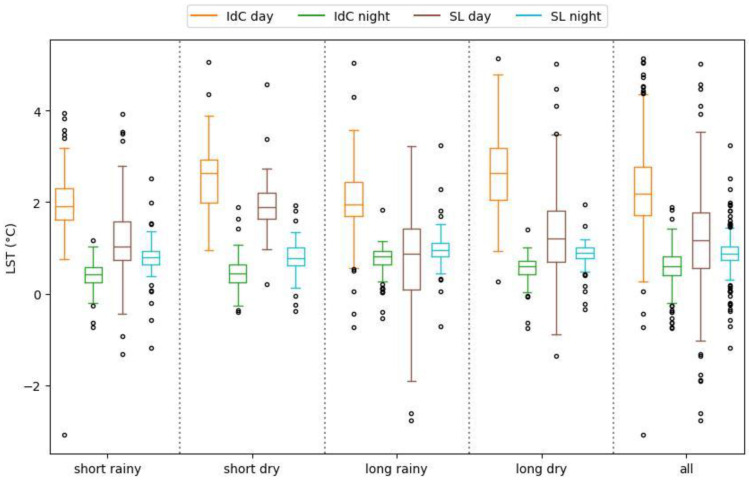
Box plot of daytime and nighttime SUHI intensity.

Figure 10 presents the box plot of daytime and nighttime SUHI intensity. On this figure, one can see that the repartition of SUHI intensity is larger during rainy seasons than dry ones. Moreover, the daytime SUHI intensity of Saint-Laurent has a longer range than that of Ile de Cayenne. This can be caused by the fact that Saint-Laurent has a greater non-urban area than Ile de Cayenne.

### 3.4. Assessment of the UTFVI

**Figure 11:**
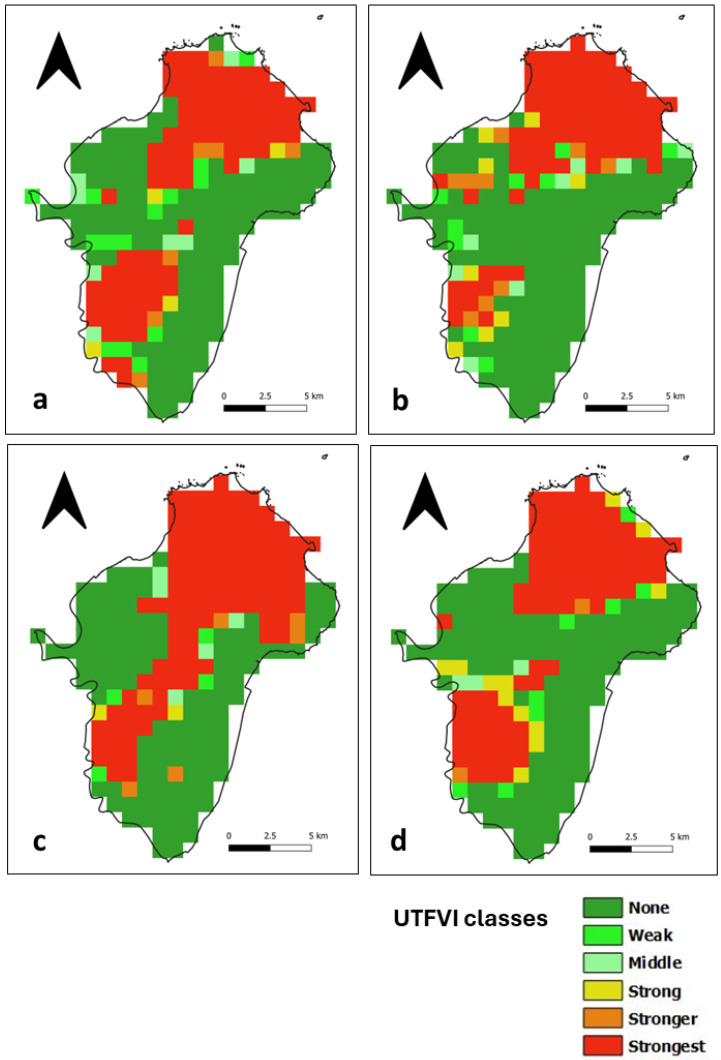
Ile de Cayenne daytime UTFVI for (**a**) 1 February 2020 (**b**) 1 April 2020 (**c**) 1 August 2020 and (**d**) 1 December 2020.

**Figure 12:**
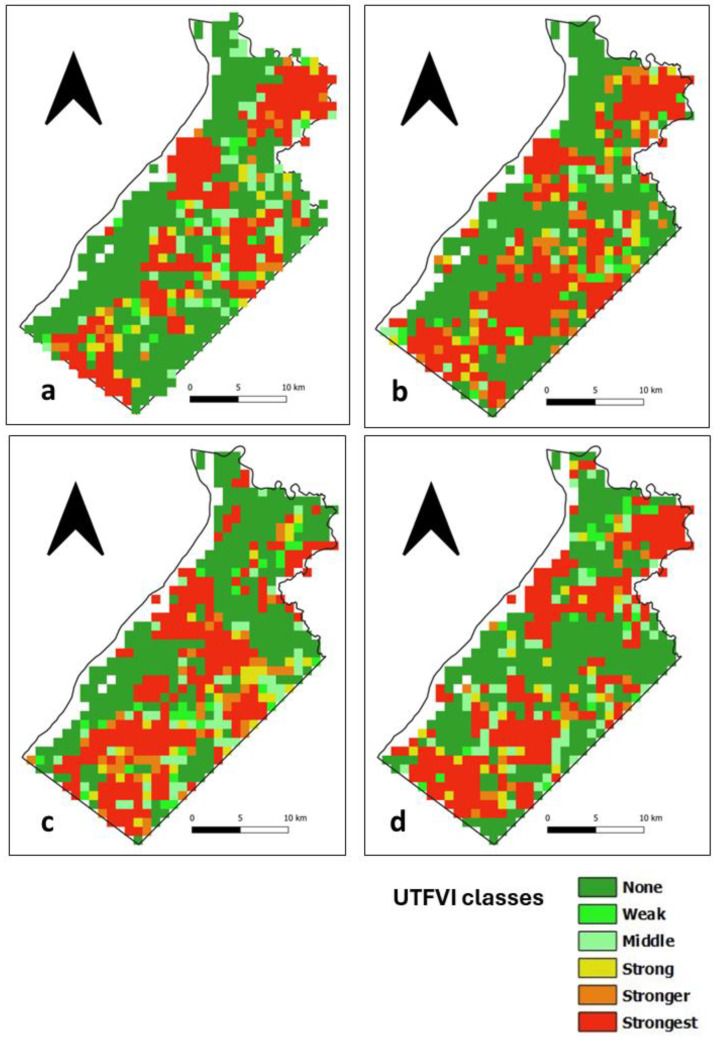
Saint-Laurent daytime UTFVI for (**a**) 1 February 2020 (**b**) 1 April 2020 (**c**) 1 August 2020 and (**d**) 1 December 2020.

The UTFVI is a SUHI index calculated from LST data that classifies the occurrence of the SUHI phenomenon into six categories, as shown in Table 1. In the daytime, areas with no SUHI are distributed throughout Ile de Cayenne (Figure 11) and correspond to vegetation cover. The presence of the strongest SUHI phenomenon corresponds to highly urbanized places in the north, where Cayenne and Remire-Montjoly are located, and in the southwest, where Matoury is located (Figure 1). The strongest SUHI phenomenon at Saint-Laurent (Figure 12) is located mainly in the west, but scattered patches are observed across the municipality.

**Figure 13:**
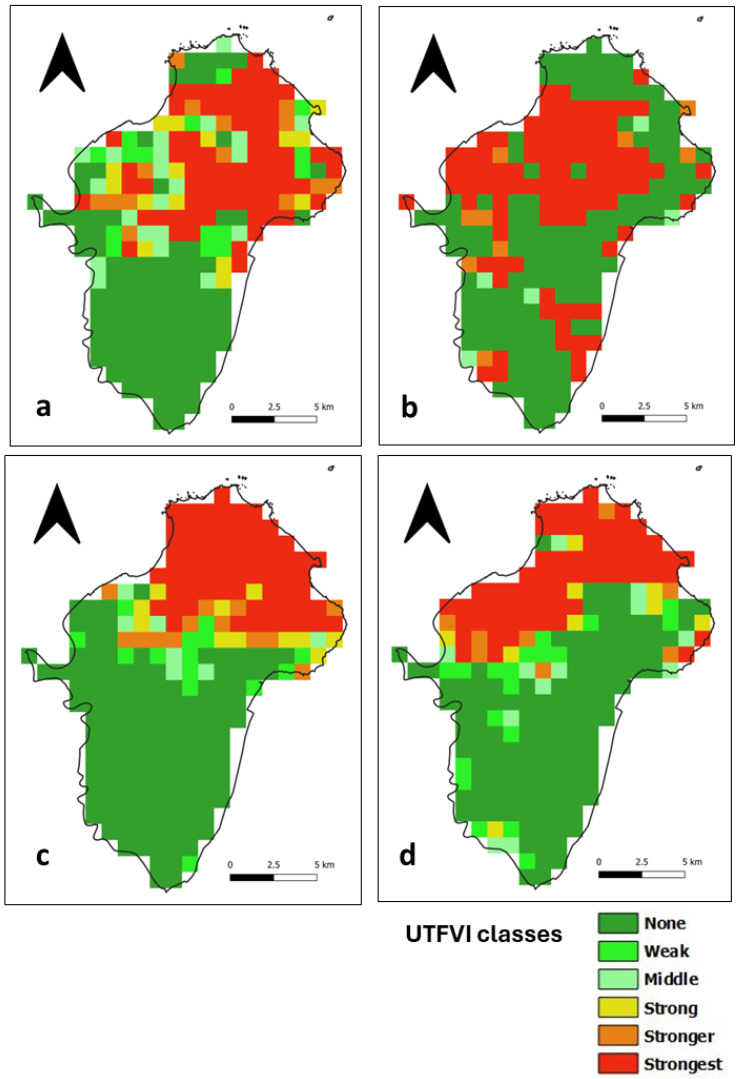
Ile de Cayenne nighttime UTFVI for (**a**) 1 February 2020 (**b**) 1 April 2020 (**c**) 1 August 2020 and (**d**) 1 December 2020.

**Figure 14:**
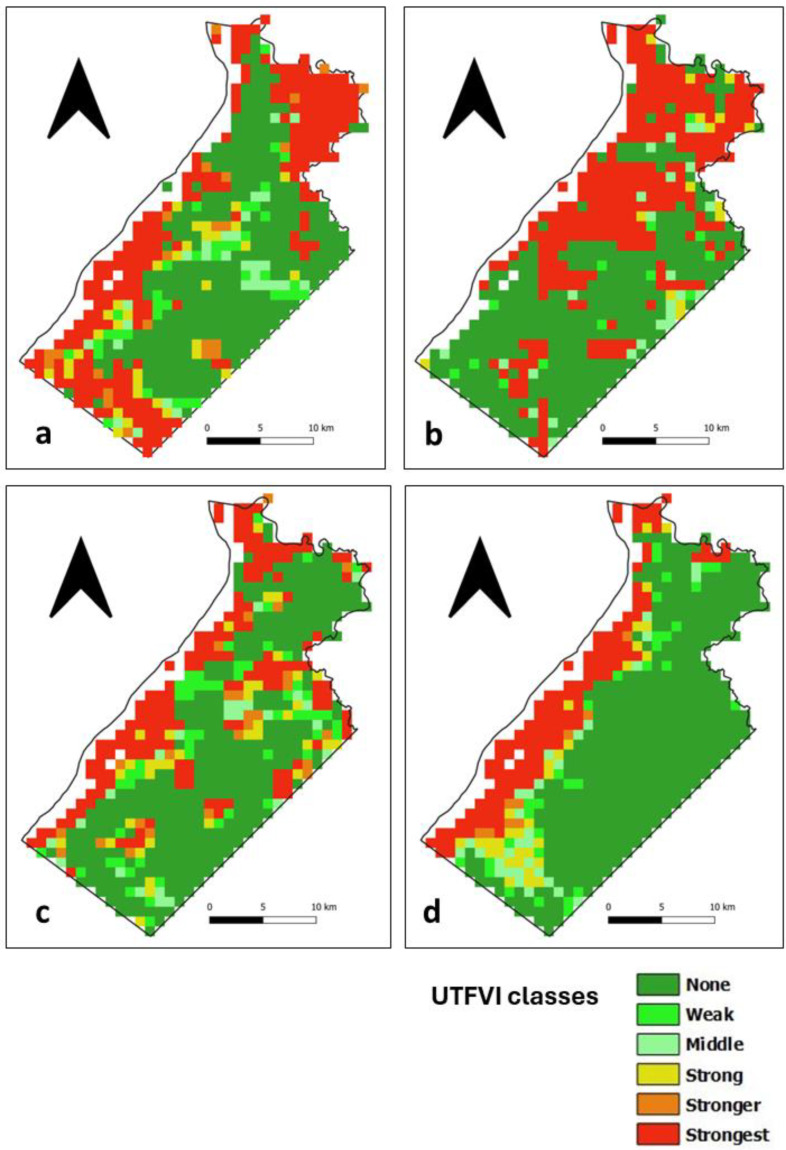
Saint-Laurent nighttime UTFVI for (**a**) 1 February 2020 (**b**) 1 April 2020 (**c**) 1 August 2020 and (**d**) 1 December 2020.

At nighttime, areas with no SUHI phenomenon at Ile de Cayenne remain dominant and are distributed throughout the region (Figure 13). The strongest SUHI phenomenon appears to remain in the north but is slightly shifting or, in some cases, expanding westward. At Saint-Laurent (Figure 14), the most interesting observation is the concentration of the strongest SUHI in the west of the municipality.

### 3.5. Relation between the SUHI and Some Related Factors

#### 3.5.1. Details on the Related Figures

Two figures are proposed for this section, and their computation is detailed hereafter.

**Figure 15:**
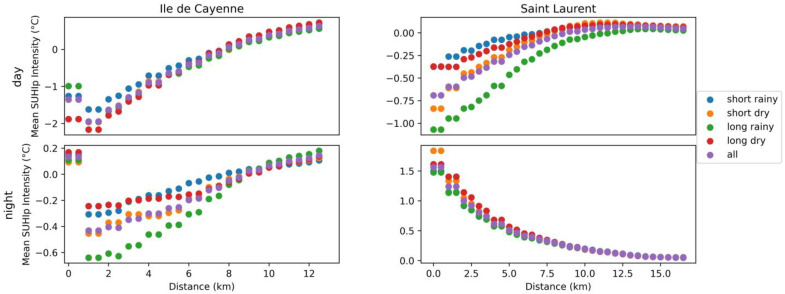
Mean LST regarding the distance to the closest water point.

Figure 15 shows the evolution of the mean of SUHI_p_ regarding the distance to the closest water point for each region, daytime, and season: four figures proposed in the first column represent the SUHI_p_ intensity evolution according to the distance to the closest water point for Ile de Cayenne and for Saint-Laurent on the second column; daytime results are presented on the first row and nighttime ones on the second row. To plot Figure 15, the distances of pixels to their closest water point are computed. Then, the range of distance values is divided into N. Next, for each distance d in this subset, the average value of SUHI_p_ values for pixels whose distance to their closest water point is between 0 and d is noted, and $\bar{{\mathrm{SUHI}}_{p}}$, is computed. 

**Figure 16:**
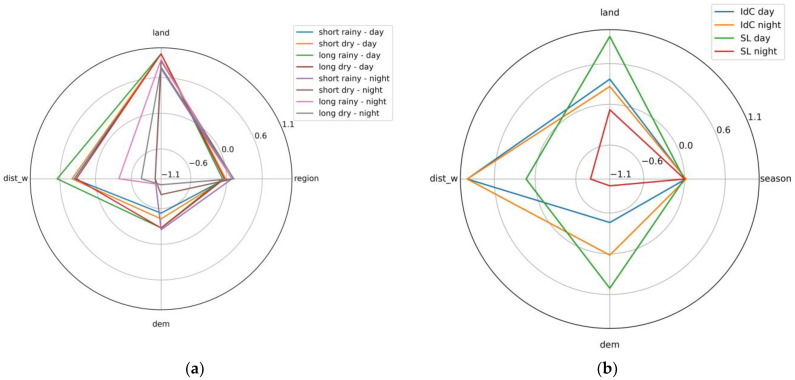
Radar diagrams of the standardized coefficients of the linear correlation between SUHI_p_ intensity and chosen factors regarding the time with the additional factor: (**a**) region and (**b**) season.

Figure 16 represents the standard value of the coefficients (i.e., the original coefficient values divided by their maximum absolute value) regarding a linear correlation between SUHI_p_ and normalized land cover, region, elevation, and distance to the water point. In addition to the factors introduced in Section 2.7, the region and season have been added. To obtain subfigure a, a linear relation has been computed between all SUHI_p_ of both regions and evaluated for each season. For subfigure b, the SUHI_p_ data are separated by region and daytime. Hence, the figure helps us understand the spatiotemporal relations. 

#### 3.5.2. Presentation of the Results

In Figure 15, the influence of the proximity of the water point is clearly observable for Ile de Cayenne at daytime and Saint-Laurent at nighttime. In the first case, the $\bar{{\mathrm{SUHI}}_{p}}$, increases with the distance, and in the second case, the $\bar{{\mathrm{SUHI}}_{p}}$ decreases with the distance. At Saint-Laurent, the greater differences are observed at nighttime rather than daytime. In all cases, the maximum $\bar{{\mathrm{SUHI}}_{p}}$, means are observed for the long dry season. During that season, at Ile de Cayenne, the $\bar{{\mathrm{SUHI}}_{p}}$, differences can reach 2.9° at daytime but are less than 1° at nighttime. In contrast, at Saint-Laurent, the difference is less than 0.5° in the daytime and 1.6° in the nighttime. The minimum $\bar{{\mathrm{SUHI}}_{p}}$, are observed for the long rainy season. Figure 16 shows a strong positive influence of the distance to water on SUHI_p_ during daytime and a negative one at nighttime; plus, this relation is almost nil at Saint Laurent during daytime. 

Figure 16 shows that the land cover has a higher influence on SUHI_p_ for Saint-Laurent in the daytime and less at Saint-Laurent in the nighttime, and its relationship with Ile de Cayenne is quite similar in terms of day and night. 

The SUHI_p_ has a negative relation with the elevation; this relation is negative at nighttime for all seasons except the short rainy one. The relation between the elevation and the SUHI_p_ values at Ile de Cayenne at nighttime is almost nil. This relation is highly positive for Saint-Laurent in the daytime. 

## 4. Discussion

In this study, we seek to understand the spatial and temporal variation of SUHI at Ile de Cayenne and Saint-Laurent. They are two of the largest urban areas in French Guiana and have important population density and economic activities. Ile de Cayenne is a coastal region, while Saint-Laurent is an inland one close to the Maroni River.

During the day and night, high LST and the strongest SUHI phenomenon were spatially distributed in built-up areas. However, at night, we noticed the disappearance of some daytime high LST and strongest SUHI patches at both Ile de Cayenne and Saint-Laurent, which suggests a faster heat dissipation than those that remained hotspots for high LST and the strongest SUHI phenomenon. 

Furthermore, for both Ile de Cayenne and Saint-Laurent, we have observed an apparent shift of high LST and SUHI toward the west. It is probably the result of wind direction as the wind in French Guiana blows from east to west. Additionally, patches with only the nighttime SUHI phenomenon could also be the result of heat island transfer facilitated by wind. According to [6], it is possible for non-heat island areas to be converted to heat island areas and low-grade SUHI to be converted to high-grade SUHI. 

Moreover, we suspect that the Maroni River (west of Saint-Laurent) and the Cayenne River (west of Ile de Cayenne) play a role in the nighttime distribution of high LST and strongest SUHI. In fact, during hot weather periods, water bodies’ temperatures are usually lower than air temperature [37,38]. As a result, their surface temperatures are often lower than that of neighboring urban areas [39]. These differences in temperatures, particularly for water bodies with a depth of at least half a meter, are the result of the large heat capacity of water in combination with its ability to transport heat away from its surface by turbulent mixing [40]. As a result, water bodies take longer to cool down, may become warmer than the air during the night, and may enhance nighttime urban heat islands [41].

Furthermore, SUHI intensity reaches its peak in the daytime. At nighttime, the intensity significantly drops below 2 °C on average. The observation that SUHI intensity is greater at daytime than at nighttime is consistent with existing studies [42]. Nevertheless, the magnitude differs both seasonally and daily. In addition, Ile de Cayenne tends to experience higher SUHI intensity than Saint-Laurent. 

Coastal regions have lower SUHI than inland regions [23]. The observations of the study are not consistent with those differences. In fact, even if Ile de Cayenne is a coastal region, it has a greater urban density than Saint-Laurent (around 593 people/km^2^ versus 9 people/km^2^). Moreover, Saint-Laurent is mostly covered by the Amazonian forest. Thus, higher SUHI is consistent in Ile de Cayenne than in Saint-Laurent. 

Different studies have shown the impact of season on the occurrence of SUHI, with summertime usually showing high SUHI intensity [43,44]. French Guiana experiences a dry season from early February to mid-March and from July to November. During the dry season, temperatures are usually high, with limited rainfall events. It has also been during these months that we have observed peaks of high SUHI intensity and maximum LST. We have also noticed that the spatial distribution of LST and SUHI was also influenced by seasonal variation. 

The proximity to the water point, the digital elevation, and the land cover also have an important influence on the LST values [35] and, therefore, on SUHI_p_. The dependency of SUHI_p_ on these factors depends on both the season and the time. As supposed, the land cover is very important; the SUHI_p_ values tend to be higher in urban areas [45]. The Maroni River mostly occupies the western part of Saint-Laurent; as water takes more time to cool down, the distance to the water point is highly negatively related to the SUHI at Saint-Laurent at nighttime. 

Urbanization in French Guiana is rapidly occurring, with some areas yet to be urbanized. As a result, findings from our study can be used by local authorities to inform future urban development in order to reduce the SUHI effect on urban dwellers. 

## 5. Conclusions 

This study aimed to assess the spatial and temporal dynamics of the SUHI in two of the largest urban areas of French Guiana, namely Ile de Cayenne and Saint-Laurent, using MODIS-based gap-filled LST data. This study is the first in French Guiana and aims to set the stage for upcoming studies. The SUHI is a broad topic that can be studied from different perspectives. 

It transpired that the high LST and SUHI phenomenon, in the case of Ile de Cayenne, was more pronounced in the north and southeast due to the presence of built-up areas. In the case of Saint-Laurent, it was more toward the north and the west. Furthermore, we observed that the intensity of SUHI was also related to season, with peaks observed during the dry season.

The study highlighted the influence of elevation and distance to the closest water point on surface urban heat islands. The Maroni River cools down at night, leading to a higher SUHI at Saint-Laurent. Plus, as Ile de Cayenne is a coastal region, it benefits from trade winds helping to cool the coastal area.

Moreover, the SUHI moves to the west from daytime to nighttime according to the direction of the wind.

Having a complete cloud-free LST time series offers the opportunity to better understand SUHI dynamics. In this study, we used MODIS-based gap-filled LST data produced by [32]. In general, the results obtained using these data are conclusive and allow us to better understand the SUHI dynamics in the French Guiana. Nevertheless, with gap-filled data, there are inevitably potential errors. We, for example, spotted a low LST of 7.7 °C at Ile de Cayenne, which we consider unusual given the geographical context of our study area. Therefore, the LST data are usable, but care should be taken when interpreting results. 

In conclusion, our study has provided valuable insights into the dynamics and variability of urban heat islands in French Guiana. We have identified significant factors contributing to the exacerbation of heat within urban environments, including land use patterns, vegetation cover, and built infrastructure. The implications of our findings are substantial for urban planning and environmental management efforts in the region. 

However, our study is not without its limitations. Our data’s spatial and temporal resolution may not capture the full complexity of urban heat dynamics. Future research should integrate more detailed climatic data (including air temperature), explore the socio-economic dimensions of urban heat exposure, and examine the impact of different mitigation strategies in diverse urban settings. Downscaling techniques may also be introduced to improve LST spatial. 

In summary, while our study has made important contributions to understanding urban heat islands in French Guiana, it also highlights the need for ongoing research and collaborative action to address this pressing environmental challenge. 

## Figures and Tables

**Table 1:** UTFVI score and associated classes [16].

UTFVI Range	SUHI Phenomenon
<0	None
0.000–0.005	Weak
0.005–0.010	Middle
0.010–0.015	Strong
0.015–0.02	Stronger
≥0.020	Strongest

**Table 2:** Comparison of average LST for Ile de Cayenne and Saint-Laurent.

Region	Season	Time	LST Max	∆LST	LST Mean Urban	LST Mean Non-Urban	SUHI Intensity
Ile de Cayenne	-	-	28.3	11.5	25	23.6	1.4
		Day	34	-	29.6	27.3	2.6
		Night	22.5	-	20.5	19.9	0.6
	Short rainy	-	27.1	10.7	24.3	23.2	1.2
		Day	32.5	-	28.6	26.6	2
		Night	21.8	-	20	21.7	0.4
	Short dry	-	28.2	12.3	24.6	23.1	1.5
		Day	34.3	-	29.5	27	2.5
		Night	22	-	19.7	19.2	0.5
	Long rainy	-	27.4	10.9	23.9	22.5	1.4
		Day	32.8	-	28.2	26.1	2
		Night	21.9	-	19.7	19	0.7
	Long dry	-	30.3	12.6	27.1	25.5	1.6
		Day	36.6	-	32	29.4	2.6
		Night	24	-	22.1	21.6	0.5
Saint-Laurent	-	-	27.1	7.7	24.1	23.1	1
		Day	31	-	27.6	26.5	1.1
		Night	23.2	-	20.7	19.8	0.9
	Short rainy	-	26.2	6.8	23.3	22.3	1
		Day	29.6	-	26.4	25.2	1.2
		Night	22.8	-	20.2	19.4	0.8
	Short dry	-	27	8.3	24	22.6	1.4
		Day	31.2	-	28.5	26.5	1.9
		Night	22.8	-	19.5	18.7	0.8
	Long rainy	-	26.4	7.7	23.5	22.7	0.9
		Day	30.3	-	26.9	26.1	0.7
		Night	22.6	-	20.2	19.2	1
	Long dry	-	28.7	8.1	25.6	24.5	1.1
		Day	32.7	-	29.1	27.8	1.3
		Night	24.6	-	22.1	21.3	0.9

## Data Availability

The datasets presented in this article are not readily available because they are the property of the funding organization. Request to access the datasets should be directed to ADEME France.
